# Microbial Control in the Processing of Low-Temperature Meat Products: Non-Thermal Sterilization and Natural Antimicrobials

**DOI:** 10.3390/foods14020225

**Published:** 2025-01-13

**Authors:** Xiaoyang Zhang, Feng Na, Min Zhang, Wei Yang

**Affiliations:** 1 College of Food Science and Bioengineering, Tianjin Agricultural University, Tianjin 300380, China; zhangxiaoyang_0_01@163.com (X.Z.); 15650226488@139.com (F.N.); zhangmin@tjau.edu.cn (M.Z.); 2 College of Basic Science, Tianjin Agricultural University, Tianjin 300380, China

**Keywords:** low-temperature meat products, non-thermal sterilization, natural bacteriostats, microorganisms

## Abstract

The safety and health of food have been persistent concerns, particularly about meat products. Low-temperature meat products refer to those that are processed at lower temperatures. Meat, rich in proteins and other nutrients, is highly susceptible to microbial contamination, leading to spoilage, particularly when processed at lower temperatures that increase storage and transportation requirements. In response to the limitations of conventional preservation methods, such as heat treatment and chemical bacteriostats, emerging preservation technologies are increasingly being adopted. These technologies aim to mitigate the negative effects of microorganisms on meat products. Non-thermal technologies and biotechnological approaches, which are low in energy consumption and energy efficiency, are becoming more prevalent. Non-thermal sterilization technology is widely applied in various food products. It maintains the original quality of food, enhances food safety, reduces energy consumption, and improves production efficiency. Biocides are extensively used in the antibacterial field owing to their high efficiency, low toxicity, and long-lasting properties. Both non-thermal sterilization technology and biocides can ensure food safety, extend the shelf life of food products, improve food quality, meet consumers’ demand for natural and healthy food, enhance market competitiveness, and play a positive role in promoting the sustainable development of the food industry. This paper provides a comprehensive review of the specific applications of biocides and non-thermal sterilization methods in food, highlighting the control parameters and their effects on microbes during low-temperature meat processing, to supply pertinent researchers with theoretical references.

## 1. Introduction

The global meat market is experiencing substantial growth and is projected to expand at a compound annual growth rate (CAGR) of 6.24% from 2024 to 2028, with the market value expected to reach USD 655.6 billion. Among the various types of meat products, low-temperature meat products have garnered significant attention due to their ability to preserve the original nutrients and flavor of the meat. These products, which include sauce and marinade goods, sausage products, ham products, and seasoned meat products, are processed at low temperatures ranging from 0 to 4 °C for marination, 70 to 80 °C for cooking, and are often produced through low-temperature steaming [1]. This production method helps retain the flexibility and moisture content of the meat, enhances its color, and keeps flavor and nutrients from being lost. However, when compared to high-temperature processed foods, low-temperature meat products are more prone to microbial contamination. Firstly, microbes can easily survive and multiply in the processing environment. The relatively low processing temperatures are inadequate to eliminate them. Secondly, microbial growth is promoted by product attributes such as high water activity, rich nutrient content, and suitable pH values. Thirdly, although low temperatures can inhibit the growth of certain microorganisms, some psychrophilic bacteria can still thrive. If the cold supply chain is not optimal, it is easy to cause microbial contamination. Moreover, the requirements for the storage environment are more stringent than those for high- temperature processed products.

Consequently, cryogenic meat products face several challenges during production and storage, such as incomplete sterilization, microbial contamination, food spoilage, shortened shelf life, and consequent economic losses.

To address this issue, various technological tools—including physical, biological, chemical, and other relevant technologies—have been investigated in the market. Physical preservation methods are generally classified into two types: thermal and non-thermal sterilization methods [2]. Traditional thermal sterilization technology has long been dominant in the food processing industry. It kills microorganisms through high-temperature treatment to ensure food safety and extend the shelf life. However, high temperatures will unavoidably degrade food quality by destroying vitamins, enzymes, and other ingredients, lowering the meal’s flavor and nutritional content. Non-thermal sterilization technologies include examples such as low-temperature preservation, irradiation, high-pressure processing, and modified atmosphere packaging. Non-thermal technologies are evolving in diverse ways to meet the processing requirements of different foods. The combined application of various technologies, such as the joint processing of high-pressure and pulsed electric fields, and the synergistic sterilization of ultrasonic and ultraviolet, has gradually become a research hotspot looking to achieve a more efficient and precise sterilization effect. These techniques offer several benefits, including lower energy consumption for sterilization, broad applicability, and better preservation of food flavor. Chemical fungicides and preservatives have long been widely used to control microbial contamination. However, chemical control methods have given rise to a series of problems, such as drug resistance, environmental pollution, and chemical residues. Biological control methods are attractive due to their broad-spectrum antimicrobial activity, hypoallergenic nature, biodegradability, and the difficulty of inducing microbial resistance. The main means of biological control are natural extracts, which are classified according to their origin as botanical, animal, or microbiological [3]. With the pursuit of healthy and green food, biological control methods have been gradually applied in food processing due to their natural, safe, and environmentally friendly features, further promoting the development of biological control methods.

With the goal of offering a scientific research foundation for the management and eradication of microorganisms in the processing of low-temperature meat products, this article primarily performs a thorough study and summary of biological and non-thermal sterilizing technologies.

## 2. Non-Thermal Sterilization Techniques

Physical sterilization technology refers to methods that do not rely on chemical additives but instead utilize physical means to eliminate or inhibit the growth of microorganisms, thereby ensuring food safety and extending the shelf life of food (Table 1).

Supercritical CO_2_ sterilization is a new type of non-thermal sterilization technology. Its sterilization process is very similar to extraction, operated mainly through a high-pressure pump to pump the CO_2_ and product into the system, which is then mixed and maintained for a period to achieve the purpose of sterilization [4]. It prevents the growth of pathogenic and spoilage microorganisms in meat and meat products at moderate pressures (7.3–50.0 MPa) and temperatures (35–55 °C) [5]. Low operating temperature and pressure, which are easy to adjust, can minimize the degradation of heat-resistant nutrients, and maintain the sensory and nutritional properties of meat and meat products. Simultaneously, this method keeps the meat’s texture, flavor, and nutrients while maximizing sterilization efficiency. However, carbon dioxide may undergo chemical reactions with some nutrients, which affects their stability and biological activity. Ferrentino et al. investigated the feasibility of supercritical CO_2_ in the inactivation of *Listeria monocytogenes* inoculated on the surface of dry-cured ham and found that all inoculated Listeria monocytogenes were inactivated under the treatment conditions of 12 Mpa, 50 °C, and 15 min and that the treatment had almost no effect on the color and organoleptic qualities of the hams. Moreover, the efficiency of supercritical CO_2_ sterilization increased with the moisture content of the meat and meat products [6]. The optimization of the sterilization process using orthogonal tests was analyzed by assessing changes in the eating quality of fresh beef. The results indicated that the optimal conditions were a sterilization temperature of 50 °C, a pressure of 14 Mpa, and a treatment time of 10 min, achieving a sterilization rate of 99%.

Modified atmosphere packaging (MAP) gases commonly used in gas-conditioned meat products include nitrogen (N_2_), carbon dioxide (CO_2_), and oxygen (O_2_). These gases help inhibit the growth and reproduction of microorganisms by reducing the concentration of oxygen and increasing the concentrations of other gases, such as carbon dioxide and nitrogen [7]. The technology employed in MAP equipment is flexible enough to accommodate the various types and specifications of food packaging. It is reasonably priced, easy to install and operate, and can quickly meet the requirements of mass production. Compared to aerobic packaging, carbon monoxide MAP (0.4% CO + 30% CO_2_ + 69.6% N_2_) demonstrated superior preservation effects on steaks, including enhanced color stability, reduced oxidation, and lower microbial counts. These advantages extended the shelf-life of marbled steaks by an additional four days [8]. In addition, colony counts, color, pH, rate of storage loss, and fat oxidation were improved when treated steaks were stored in the specified headspace gas composition (50% O_2_, 40% CO_2_, and 10% N_2_) [9].

Ultra-high-pressure sterilization technology uses different media to apply a static pressure ranging from 100 to 900 Mpa. The pressure is applied within an ultra-high-pressure chamber containing pre-packaged food, and the extremely high pressure disrupts microbial cell membranes, nucleic acids, and other cellular components. This process produces a destructive effect that inhibits the activity of intracellular enzymes, prevents DNA replication, and leads to the loss of survival enzymes, ultimately killing microorganisms [10]. Ultra-high-pressure equipment has high technical requirements and incurs relatively high equipment costs. Since UHP technology does not cause a sharp temperature rise, it can ensure food safety while retaining nutrients such as proteins, amino acids, and vitamins, as well as organoleptic qualities such as the color and flavor of the food. However, UHP technology also faces some challenges. High-pressure equipment is difficult to manufacture and maintain. For large-scale production, multiple high-pressure devices are required to work in tandem, which increases the complexity and cost of the system. Beatriz et al. found no significant differences in color, texture, or tenderness of beef treated with hyperbaric pressure compared to untreated samples. However, the shelf life of beef treated with hyperbaric pressure was extended to 42 days under refrigerated conditions (4 °C), considerably prolonging the consumption period [11].

Irradiation sterilization is a highly effective food preservation technology that treats foodstuffs through ionizing radiation to extend their shelf life and maintain their quality. This method effectively inhibits or destroys pathogenic microorganisms, spoilage bacteria, yeasts, and molds in food products, thereby ensuring safety and prolonging storage time. Irradiation equipment is usually complex and expensive. It requires specialized facilities, and specialized personnel, and incurs high construction and maintenance costs. However, it can bring high economic benefits for some high-value-added food products or those that require long-term storage and transportation. The mechanism of action involves two primary pathways: direct and indirect. In the direct pathway, radiation energy directly damages critical biomolecules such as DNA, RNA, lipids, and proteins in microorganisms, suppressing their growth and reproduction. The indirect pathway involves the irradiation of water molecules, producing reactive substances that interact with biomolecules through redox reactions, ultimately disrupting the structure and function of microorganisms. Among these, indirect effects play a significant role in causing DNA damage [12]. The Hygienic Standard for Irradiated Cooked Livestock and Poultry Meat stipulates that the overall average absorbed dose for cooked livestock and poultry meat should not exceed 8 kGy. Unlike conventional radiation, electron beam irradiation does not rely on radioisotopes to produce ionizing radiation. It achieves microbial inactivation by accelerating electrons to nearly the speed of light in a vacuum environment at energies ranging from 0.15 to 10 mega-electron volts. This process generates a high-energy electron beam that disrupts molecular and atomic bonds in microorganisms within food. The resulting free electrons and ions damage microbial DNA structures, denature membrane proteins, and inactivate enzymes, leading to the loss of reproductive capability and essential physiological functions in bacteria [13].

Microwaves are typically defined as electromagnetic waves with frequencies ranging from 300 MHz to 300,000 MHz and corresponding wavelengths between 1 mm and 1 m. This technology is a non-thermal sterilization technology, with the help of a 650 W microwave, 5 min of treatment can eliminate Candida albicans and other germs, and more than 7 min of treatment can fully eliminate Staphylococcus aureus. Microwave technology features a fast-processing speed. It can heat and sterilize food in a short period, improve production efficiency, and is suitable for large-scale continuous production. Compared with traditional methods, the nutrient loss is relatively small. In addition, microwave equipment occupies a relatively small area. Its installation and commissioning are relatively simple, and it can be quickly adapted to different production sites and production process requirements.

Ice temperature insulation technology belongs to the third generation of preservation technology [14], offering unique advantages as technology controls the temperature of the food to be above the freezing point and close to 0 °C. This range is referred to as the “ice temperature zone” or simply “ice temperature”. Under ice temperature conditions, foods remain near a frozen state but do not freeze completely. This temperature control strategy satisfies the minimum requirements for maintaining food’s physiological activity while effectively slowing microbial growth and reducing endogenous enzyme activity, thereby extending the food’s freshness period [15]. For some fresh food products with high-quality requirements, such as fruits, vegetables, and meat, the use of ice temperature technology can significantly increase the market value of the products and reduce losses. However, the stringent precision requirements of refrigeration equipment for ice temperature technology limit its practical application, often making it difficult to achieve the desired freshness preservation effects. Consequently, this technology is frequently combined with natural antimicrobial agents to enhance its performance.

**Figure 1:**
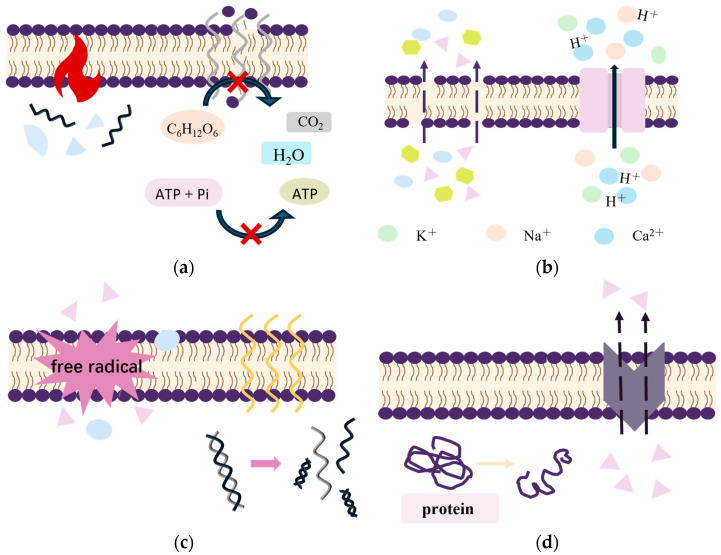
The main physical antibacterial mechanisms of action. (**a**): microwave (**b**): pulsed electric field (**c**): irradiance (**d**): oppressive.

The following are the antibacterial mechanisms of four representative physical methods (Figure 1).

**Table 1:** Effects of physical techniques on microorganisms.

Physical Technology	Technical Parameters	Culture Condition	Target of an Action	Consequences of Action		Medium of Action	Bibliography
Supercritical CO_2_	12 MPa, 50 °C, 15 min	37 °C, 48 h	*Listeria monocytogenes*	*Listeria monocytogenes* inactivation 10^7^ CFU/g.	-	Cured ham	[6]
	121 MPa, 50 °C, 5 min	-	mesophilic aerobic bacteria, psychrophile, Lactic Acid Bacteria, yeasts, mold, and *Escherichia coli*	Reductions of 3.0, 1.6, and 2.5 log CFU/g were observed for mesophilic aerobic bacteria, psychrophilic bacteria, and lactic acid bacteria, respectively. Meanwhile, yeasts, molds, and *Escherichia coli* were reduced to levels below the detection limit.	-	Hams	[16]
Air conditioning	30% CO_2_/70% N_2_	−1 ± 0.1 °C	*Pseudomonas* spp., *Lactic Acid Bacteria*, and *Enterobacteriaceae*	Significantly inhibited the growth of *Pseudomonas* spp.	Extended storage period to 31 days.	Chicken meat	[17]
	25% CO_2_, 35% N_2_	4 °C	*Pseudomonas* spp., *Lactobacillus*, and *Carn obacterium*	-	Maximum shelf life of 9 days.	Frozen boneless beef	[18]
	50% O_2_/40% CO_2_/10% N_2_	2 °C	*Pseudomonas* spp., Lactic Acid Bacteria, *Enterobacteriaceae*	-	Extends steak shelf life to 20 days.	Steak	[9]
Ultra-high pressure	600 MPa, 6 min, 31 °C	4 °C	Lactic Acid Bacteria, *enteric bacteria*	After 60 days of pressurization, the counts of lactic acid bacteria were reduced by 6 logs compared to the counts in the blank samples.	Shelf life can be extended to at least 120 days.	Sliced corned beef and ham	[19]
	600 MPa, 20 °C, 180 s	4 °C	*Listeria monocytogenes*, *staphylococcus*, *Streptomyces thermophilus*, *coliform*, yeasts, mold	Remains non-detectable for 95 days after pressure treatment.	The shelf life is significantly extended from approximately 45 to 50 days under refrigeration to at least 98 days.	Ready-to-eat (RTE) meat (low-fat pastrami, Strasbourg beef, export sausages, and Cajun beef)	[20]
	310 MPa, 324 MPa, 345 MPa, 1 min, (25 ± 2) °C,	4 °C	Aerobic bacteria (ATC)	Reduction in total ATC 1.37 log_10_ CFU g.	Shelf life up to 42 days.	Beef	[11]
Irradiation	10 kJ/m^2^	20 °C	*Pseudomonas*	The populations of total aerobic bacteria were significantly reduced by 1.76 log CFU/g.	Shelf life of 60 days.	Beef jerky	[21]
	0.5, 1, 2, 5, 10 kGy.	4 °C	*E. coli O157:H7*, *S. Typhimurium*	For *E. coli O157:H7* and *S. Typhimurium*, single or repeated irradiation at 0.5 kGy resulted in complete inactivation.	-	Fish products	[22]
	3 kGy	4 °C	*Escherichia coli*, *Listeria monocytogenes*	Effectively eliminates these bacteria over 4 log and 3 log units, respectively.	Safe storage for 12 days	Raw beef sausage	[23]
	6. 0 kGy	4 °C -	*total bacterial count*, *coliforms*, and *Staphylococcus aureus*	Total bacterial count, coliforms, and *Staphylococcus* aureus were reduced to national food safety standards.	6. 0 kGy prolonged the shelf life to 10 days at least.	Spiced beef	[24]
	2 kGy	4 ± 0.5 °C	*Salmonella*, *S. typhimurium*	*Salmonella*, *S. typhimurium*, and total bacterial populations were reduced by 5, 3, and 3 log, respectively, at 2, kGy doses, 4 °C, and 45 days *Salmonella* levels were reduced to zero.	-	Beef sausage	[25]
	7.5 kGy	3 ± 1 °C	*Staphylococcus aureus*, *Serratia marcescens*, *Enterobacter cloacae*	Microbial community 7.23 log CFU/g to 1.56 log CFU/g.	Extended shelf life by two months.	Smoked pearl chicken	[26]
microwave	896 MHz, 3 °C, 7 kw, 2 min	37 °C	*the total plate count*	On the seventh day, the total plate count (TPC) of intermediate moisture saury was 3.36 log CFU/g.	-	Intermediate moisture pacific saury	[27]
Super chilling	-	−2 ± 1 °C	*Total aerobic bacteria*	TAB < 7 log CFU/g for 14 day subcooled samples.	Subcooling can extend the shelf life of beef by at least two times compared to refrigeration.	Beef cuts	[28]
	-	−3 °C	Total Viable Count	When stored for 6 days, the total viable count (TVC) values of cattle and buffalo tripe were 5.92 and 5.97 log values, respectively, which did not exceed the limit value.	Ultra-cold stored tripe has a shelf life of up to 6 days, double that of refrigerated.	Tripes	[29]
	-	–1 °C	total viable count (TVC), *E. coli*, *V. parahaemolyticus*	On day 28, TVC increased to <300 CFU/g, and levels of *Vibrio parahaemolyticus* (<3.0 MPN/g) and *E. coli* (<18 MPN/100 g) remained extremely low.	Subcooled storage can extend shelf life up to 21 days.	Crassostrea gigas	[30]
Pulsed light (PL)	11.9 J/cm^2^	4 °C	*Listeria monocytogenes*, *S. typhimurium*, *Salmonella*	1.5 logcfu/cm^2^ to 1.8 logcfu/cm^2^.	-	Salami and pork tenderloin	[31]
Low concentration acidic electrolytic water (LCAEW)	Spray meat samples 120 s, 0.1% NaCl, 10 min	4 °C	Yeasts, Mold, *psychrophile*	Decrease in total microorganisms by 3.25 logs, yeasts and molds by 2.68 logs, and total chilled bacteria by 3.10 logs.	-	Pork	[32]

Note: - means not detailed in the article.

## 3. Biological Control Technology

**Figure 2:**
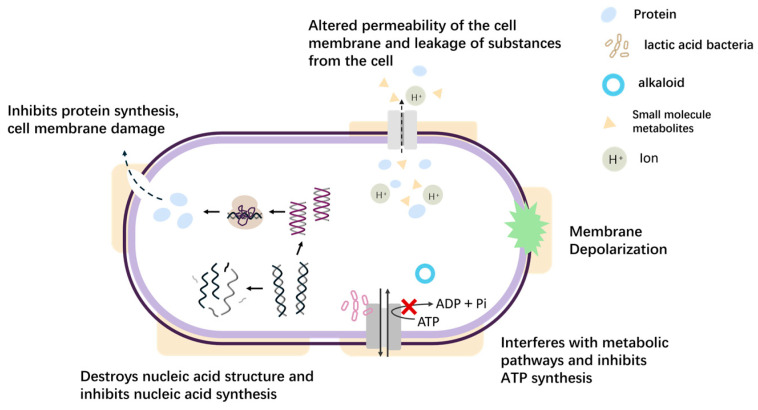
The main antibacterial mechanisms of action of natural antibacterial agents.

Natural antimicrobials, mainly of animal, plant, and microbial origin, play an important role in inhibiting the growth of microorganisms in meat products. These antimicrobials function through two primary mechanisms (Figure 2). The first mechanism disrupts the structure of the microbial cell by altering the permeability and fluidity of the cell membrane and compromises the integrity of the cell wall. This disruption impairs the normal metabolism of substances within the cell [33], limits the microorganism’s access to essential nutrients, and ultimately causes growth arrest. The second mechanism involves interference with microbial activity, as natural antimicrobials hinder microbial migration and adhesion, thereby inhibiting the synthesis of proteins and genetic material. This interfering effect hinders the normal growth process of microorganisms. Ultimately, this interference results in cell death.

Although natural antimicrobials possess certain advantages in meat production, such as high safety and diverse antimicrobial mechanisms, they encounter numerous challenges in large-scale applications. These challenges include high costs, issues related to stability and activity, problems of allergy and resistance, as well as difficulties in standardization and quality control. To enhance the practicality of natural antimicrobials in large-scale meat production, further research and innovation are essential. For example, developing more cost-effective extraction and purification methods to enhance the stability and potency of natural antimicrobials; establishing a comprehensive set of quality control guidelines and criteria to ensure their safe and efficient application in meat production; and conducting allergy and toxicity tests, and devising methods for allergen labeling.

Only through continuous practical advancements can natural antimicrobials be more extensively applied in large-scale meat production and realize their potential value.

### 3.1. Bacteriostatic Agents of Animal Origin

Animal-derived bacteriostatic agents are substances with antimicrobial properties extracted from animals (Table 2).

Chitosan is a long-chain polymer consisting of glucosamine and N-acetylglucosamine linked by a β-1,4-glycosidic bond, which has good antibacterial activity. According to Vaz et al. [33], chitosan can achieve bacterial inhibition in two main ways. Firstly, chitosan will be adsorbed to the negatively charged cell wall, resulting in the rupture of the cell wall, and the bacteria will lose the important protective barrier and thus be inactivated; secondly, chitosan will flocculate with anionic cell cytoplasm of the bacteria and affect its metabolic process, thus generating the effect of bacterial inhibition [34]. Chitosan’s broad-spectrum antibacterial activity is beneficial for meat preservation [35]. Its inhibitory effect is more pronounced against gram-positive bacteria than gram-negative bacteria. However, its efficacy decreases when the pH exceeds 6.5, with optimal inhibition observed at a pH range from 5.0 to 5.5. Chitosan exhibits significant antimicrobial properties and excellent film-forming abilities, making it widely used in food preservation as an effective and sustainable material for developing antimicrobial films for food packaging. Its incorporation enhances both the antimicrobial efficacy and moisture-regulating properties of the composite films. Furthermore, the biodegradability and non-toxicity of chitosan make it an optimal choice for creating environmentally friendly and food-safe packaging solutions, aligning with the growing demand for sustainable alternatives in the food industry [36].

Propolis is a natural substance produced by bees from plant resins combined with their palpal and wax gland secretions and is rich in biological and pharmacological activities [37]. It contains a variety of chemical components, including flavonoids, aldehydes, phenols [38], and other bioactive substances that disrupt the cell wall and membrane structure of bacteria, resulting in bacterial death [39].

Antimicrobial peptides (AMPs) are a class of important natural immune defense molecules produced by living organisms with broad-spectrum antimicrobial activity [40]. They are capable of rapidly killing both gram-negative and gram-positive bacteria, as well as fungi and other microorganisms. Characterized by low resistance, low toxicity, biodiversity, and direct aggressiveness, AMPs primarily kill bacteria by disrupting the integrity of the bacterial cell membrane. In addition, some antimicrobial peptides (AMPs) exert their antimicrobial effects through non-membrane mechanisms, such as interfering with intracellular metabolism, inhibiting protein synthesis, and binding to DNA [41].

Ichthyosperm is an alkaline protein extracted from the mature spermatozoa of fish, exhibiting a broad spectrum and highly efficient bacteriostatic properties. It demonstrates significant inhibitory effects on pathogenic bacteria, including *Listeria monocytogenes*, *Staphylococcus aureus*, *Vibrio parahaemolyticus*, *Salmonella*, and *Pseudomonas aeruginosa*. Notably, its bacteriostatic effect is more pronounced in foods rich in sugars and proteins but low in fat content. The inhibitory mechanism primarily occurs through interactions with cell wall membranes, genetic material, or functional protein [42].

**Table 2:** Effects of animal-derived antibacterial agents on microorganisms.

Antibacterial Agent	Dosages	Culture Condition	Target of an Action	Consequences of Action		Medium of Action	Bibliography
Chitosan	0.5%, 1%	4 °C	Lactic Acid Bacteria, *Pseudomonas* spp.	LAB counts were approximately 1 and 1.5 log CFU/g lower than control samples, respectively.	Acceptable level within 21 days	Pork sausage	[43]
	1.0%	4 °C	Yeasts, mold, Lactic Acid Bacteria	The maximum colony size was 3 log CFU/g at 18 days.	Shelf life extended from 7 to 15 days	Pork	[44]
	1.0%	4 °C	*Staphylococcus aureus*, *Pseudomonas*, *Proteus vulgaris*, and *Escherichia coli*	3.9 and 4.1 log CFU/g, respectively, until the end of the storage period.	Effectiveness for 20 days	Pork sausage	[45]
Propolis	0.5%, 1.0% 2.0% (*w*/*v*)	4 °C	total mesophilic aerobic	The total mesophilic aerobic bacteria on day 49 for 0.5%, 1%, and 2% treatments were 5.41, 3.84, and 3.73 log CFU/g, respectively.	-	Tuscan sausage	[46]
	0.15 mg/mL	4 °C	*Clostridium*	The addition of propolis reduced the number of non-toxic *Clostridium* difficile by 3 log CFU/g on day 5.	-	Fermented meat sausage	[47]
α137-141	0.5% (*w*/*w*).	4 °C	coliform	The coliform count was 5.07 ± 0.09 log CFU/g and had the slowest growth in viable colony counts.	Inhibits microbial growth for 14 days under refrigerated conditions	Beef	[48]

### 3.2. Plant-Derived Bacteriostatic Agents

Plant-derived antimicrobials refer to natural compounds with antimicrobial properties that are extracted from plants [49], commonly encompassing essential oils, alkaloids, flavonoids, terpenoids, polyphenols [50], and polysaccharides (Table 3).

Commonly studied polyphenols include tea polyphenols and carvacrol. Tea polyphenols refer to a group of non-toxic and odorless natural antibacterial compounds extracted from tea, comprising catechins, flavones, anthocyanins, and phenolic acids. The antibacterial activity of tea polyphenols operates through three primary mechanisms. First, they specifically coagulate proteins, thereby disrupting their normal physiological functions; second, tea polyphenols can bind with bacterial DNA, which inhibits bacterial growth and reproduction; and third, tea polyphenols can disrupt the structure of the cell membrane of the bacteria, which can contribute to the inactivation of the bacteria [51,52,53].

Carvacrol, a major constituent of essential oils such as oregano and thyme, can disrupt the integrity of bacterial cell membranes and cell walls, causing changes in cell membrane permeability and bacterial morphology [54] leading to bacterial death. Additionally, carvacrol can synergize with thymol to penetrate microbial cell membranes, interact with intracellular targets, and produce a bactericidal effect [55].

Plant essential oils are a class of aromatic plant-derived secondary metabolites characterized by their distinctive odors. Their distribution within plant tissues varies depending on the species. Due to their unique hydrophobic properties and mechanisms of action, essential oils exhibit antimicrobial activity by disrupting microbial cell membrane structures and permeability, as well as interfering with the cellular metabolism. These actions ultimately lead to the loss of cellular components and functions [56]. Essential oils, such as those extracted from oregano, rosemary, thyme, and cinnamon, are widely applied in the preservation of meat products. The addition of essential oils from parsley seeds and rosemary increases the production of volatile aroma compounds and adds flavor to the food while improving the safety and quality of turkey meat to prolong its freshness [57].

Natural spices such as garlic, peppercorns, cinnamon, cloves, and thyme are widely used in the processing of traditional cooked meat products in China. These spices are rich in bioactive compounds such as allicin, polyphenols [58], flavonoids, and functional fatty acids [59], which exhibit antibacterial, antiseptic, and antioxidant properties [60]. Ayfer et al. who studied the bacteriostatic activity of cinnamon and other extracts found that its ethyl acetate, acetone, and chloroform extracts were bacteriostatic against *Listeria monocytogenes* [61]. The primary antibacterial compound in clove is eugenol, while the main active ingredient in cinnamon is cinnamaldehyde. The ethanol extract of rosemary contains ursolic acid, the components of which need to be investigated further. Cinnamaldehyde extracted from cinnamon has demonstrated fungal and bacterial inhibitory properties [62,63]. It can be observed that each of these spices contains its unique antibacterial component.

**Table 3:** Effects of plant-derived antibacterial agents on microorganisms.

Antibacterial Agent	Dosages	Culture Condition	Target of an Action	Consequences of Action		Medium of Action	Bibliography
Peppermint Essential Oil	0.5%, 1%, 1.5%, *v*/*w*	4 °C	*Pseudomonas* spp.	12 day bacterial population decreased by 1–4 log CFU/g.	-	Camel meat	[64]
Freeze-dried Allium sativum along with its spray-dried microencapsulated essential oil	20%	4–8 °C	Total aerobic mesophilic flora (GAMT), *E. coli*, Sulfite-reducing anaerobes (CSR)	On the sixth day of storage, the GAMT was 6.4 ± 0.4 log CFU/g. *E. coli*, and sulphite-reducing anaerobes (CSR) were not detected.	The shelf life of minced meat with satisfactory quality (x ≤ m) is extended by 4 days.	Pork	[65]
*Eucommia ulmoides* male flower extract (EUMFE)	40, 80 mg/mL	4 °C	*Staphylococcus aureus*	The number of *Staphylococcus aureus* strains in cooked beef was significantly reduced after treatment (*p* < 0.05).	-	Beef	[66]
Freeze-dried pomegranate peel nanoparticles (LPP-NP)	1%	4 ± 1 °C	psychrophile	A reduction of 2.91 log CFU/g in colony count was observed compared to the control group.	Storage for up to 15 days.	Beef meatballs	[67]
Extraction and encapsulation of Laurus nobilis leaf extract with nano-liposome	-	4 ± 1 °C	*Escherichia coli*, *Staphylococcus aureus*	No *Escherichia coli* was observed in the 1500 ppm nanocapsule extract on day 8.	Storage for 16 days.	Spray ground beef	[68]
Addition of microencapsulated jabuticaba extract	20 g/L (2%)	1 ± 1 °C	*Staphylococcus aureus*, *Escherichia coli*	Microbiological reduction of more than 1 logCFU.	Storage for 15 days.	Pork sausage	[69]
Rosa Canina extract	0.1%	4 ± 1 °C	-	-	The shelf life was extended by one week.	Instant beef cocktail sausage	[70]
Black quinoa	2.5%	4 °C	MAB, LAB	During the 21 days of storage, MAB and LAB count remained below 4 log CFU/g and 3 log CFU/g, respectively.	-	Bologna-type sausages	[71]

### 3.3. Bacteriostatic Agents of Microbial Origin

Microbial-derived antimicrobial agents are a class of compounds produced by microorganisms that can inhibit or kill other microorganisms. Some antimicrobial agents are secondary metabolites produced by microorganisms, including antibiotics, antifungal agents, and antiviral agents. Others are antimicrobial peptides produced through microbial fermentation engineering (Table 4).

Nisin, also known as lactococcin, is a natural bioactive antimicrobial peptide produced by the fermentation of Lactococcus lactis [72]. Nisin is the only bacteriocin authorized for use as a food additive worldwide, due to its potent inhibitory effect on various gram-positive bacteria, including both food spoilage and pathogenic bacteria. Its mechanism of bacterial inhibition has two aspects, one is the perforation and destruction of the cell membrane, and the other is the reaction with the sulfhydryl group on the glycolipid synthase to inhibit its activity, preventing the synthesis of the cell wall, which leads to the death of microorganisms. Nisin is considered a safe and highly effective antimicrobial agent [73]. Since nisin acts mainly through the cell membrane and gram-negative bacteria have thin cell walls, resulting in their insensitivity to nisin, it is often used in combination with other bacteriostatic agents [74,75]. Nisin-loaded bacteriocins (NBCNs) significantly inhibited the growth of Lactobacillus rhamnosus and Leuconostoc mesenteroides in vacuum-packed beef [76]. However, it is particularly effective against gram-positive bacteria, especially spoilage organisms such as *Lactobacillus*, *Staphylococcus*, *Clostridium botulinum*, *Listeria monocytogenes*, and other spoilage bacteria [77].

ε-Polylysine is a natural cationic antimicrobial agent with a broad spectrum of bacterial inhibition, demonstrating high stability and resistance to decomposition or inactivation even at elevated temperatures and in acidic or alkaline environments. The mechanism of bacterial inhibition is widely believed to involve the damage of cell membrane systems, intracellular genetic material, enzymes, or functional proteins by ε-polylysine. ε-Polylysine demonstrated significant antimicrobial activity against common foodborne pathogenic bacteria, such as *Salmonella* [78], *Staphylococcus aureus* [79], and *Listeria monocytogenes* [80], as well as inhibiting other spoilage organisms in food.

Antimicrobial enzymes are primarily categorized into two groups: lysozyme and oxidoreductases. Glucose oxidase exerts its antimicrobial effect primarily through the generation of hydrogen peroxide (H_2_O_2_), which exhibits cytotoxicity, as well as the formation of gluconic acid, which lowers the pH and contributes to bacteriostasis [81]. The active substances generated in the reaction catalyzed by the lactoperoxidase class can destroy some important proteins in the cell to achieve bacteriostatic purposes. Lysozyme is an alkaline globulin composed of one or more polypeptide chain, with its primary site of action being the cell wall of microorganisms [82].

Lactic acid bacteria are good alternative strains for biocontrol agents due to their ability to produce metabolites such as organic acids, bacteriocins, and other bacteriostatic substances, which serve as active antimicrobial components in food [83]. Danielski et al. demonstrated that *Carnobacterium maltaromaticum* inhibits the growth of *Listeria innocua* in tray-packed cooked ham without affecting the product quality during storage [84].

**Table 4:** Effects of microbiological-source antibacterial agents on microorganisms.

Antibacterial Agent	Dosages	Culture Condition	Target of an Action	Consequences of Action		Medium of Action	Bibliography
bacteriocinogenic Lactobacillus curvatus UFV-NPAC1	12.5, 6.25 mg/g	7 °C	*Listeria monocytogenes*	*Listeria monocytogenes* reductions ranged from 1.0 to 3.0 log CFU/g.	Storage period 10 days	Sausages	[85]
Lactic acid bacteria	*L. sakei* subsp. *carnosus*/*L. sakei* + *S. xylosus* (1/2 ratio)	4 ± 2 °C	*B. thermosphacta*	The 12 day colony count did not exceed the 3.1 log CFU/g level.	Shelf life extended to 12 days	Beef	[86]
	*Lactobacillus acidophilus*, *Lactobacillus casei*, *Lactobacillus rhamnosus combination marinade* (ML)	4 °C	*Escherichia coli*, *Listeria monocytogenes*, and *S. typhimurium*	*Escherichia coli O157:H7*, *Listeria monocytogenes*, and *S. typhimurium* colony counts in the 0.7–2.7, 2.1–3.3, and 0.8–2.0 log CFU/g.	-	Beef	[87]
Calcium propionate (CaP) and tea polyphenols (TPs)	0.3% CaP + 0.03% TPs	4 °C	TVC	TVC was consistently less than 4% during the 12 day storage period.	Shelf life is extended by at least 4 days.	Stewed beef	[88]

## 4. Control of Microorganisms in Meat Products by Fencing Techniques

Hurdle technology is a food preservation method that extends the shelf life and maintains the quality of food products through the combined action of multiple preservative techniques, including but not limited to ultraviolet (UV) radiation, irradiation, modified atmosphere packaging, and other physical and biological means, to achieve a synergistic effect (1 + 1 > 2’) [89].

### 4.1. Composite Inhibitors and Composite Coatings

The combined use of multiple natural antimicrobial agents can exert a synergistic effect and improve the antimicrobial effect (Table 5), which is important for improving the quality and extending the shelf life of food products such as meat products [90]. The principle underlying this synergistic effect is based on the distinct mechanisms of action of different antimicrobial agents on microbial cells. For example, some agents may disrupt the cell wall, while others may interfere with cell membrane function or inhibit protein synthesis. Edible composite films prevent protein denaturation and water loss, improve food quality, and incorporate antimicrobial agents that act on the food, forming a protective barrier against moisture and gas infiltration. This barrier not only inhibits the growth of harmful microorganisms [91] but also preserves freshness without altering the original flavor and texture of the food.

### 4.2. Physical Technology Linkage

High-pressure treatment, UV irradiation, pulsed electric fields, cold plasma, and other non-thermal physical sterilization methods have become effective substitutes or additions to conventional heat treatment due to their high efficiency and quick microbial killing in low-temperature settings. Multiple physical processes working in concert can damage germs’ cellular structures more thoroughly, increasing the bactericidal efficiency (Table 6). High-pressure treatment combined with UV light, for instance, enhances the UV light penetration and microbe mortality, particularly for some bacteria resistant to radiation or pressure. The integrated approach can better regulate the nutrient content and organoleptic qualities of food products than individual physical or chemical treatments can, preventing nutrient loss and flavor alteration brought on by excessive heating. The ongoing advancement of non-thermal physical sterilization technology also opens the door to its widespread use and encourages the food processing sector to grow in a way that is safer, more effective, and more ecologically friendly.

### 4.3. Synergistic Treatment with Biotechnology and Physical Technology

The combination of physical inhibition and bacteriostatic agents offers significant complementary advantages over single-agent use (Table 7). The two mechanisms of action are synergistic, with the physical destruction of the microbial structure, while the bacteriostatic agent more readily penetrates the cell, facilitating multi-target inhibition of microorganisms and delaying the emergence of microbial resistance. Furthermore, this combined approach can reduce the required dosage of bacteriostatic agents, thereby cutting costs and minimizing the potential adverse effects on human health or the environment associated with high-dose usage.

When combined with a high-voltage electrostatic field (HVEF), the antibacterial effect of cinnamon essential oil (CEO) was significantly enhanced. The required antibacterial concentration of the CEO in this combination was only one-quarter to one-third of that needed when used alone. Both CEO and HVEF treatments altered the permeability of microbial cell walls and compromised the integrity of cell membranes, leading to changes in membrane protein structure and an increase in intracellular reactive oxygen species (ROS). However, the combined treatment of HVEF and CEO (HVEF + CEO) exhibited a significantly stronger antimicrobial effect than either treatment alone. Furthermore, high-voltage electrostatic fields have been shown to improve the effectiveness of CEO in preserving the freshness of minced pork, extending its shelf life from 3 to 7 days without compromising its original organoleptic qualities [127]. The combination of radiofrequency (RF) heating and antimicrobial agents enhances bactericidal efficacy. In 2004, Al-Holy et al. [124] developed a combined RF pasteurization process (65 °C) and 500 IU/mL of Streptococcus lactis, which was found to completely inactivate *Listeria monocytogenes* in sturgeon and salmon caviar products within 2 min of reaching the target temperature, without significantly affecting food quality. The combination of supercritical CO_2_ and additives can enhance bacterial inhibition, for example, by incorporating substances such as coriander essential oil and rosemary into the supercritical CO_2_ sterilization process. Meat products treated with radiation can break chemical bonds and kill bacteria in order to accomplish antibacterial goals [128]. These changes accelerate the oxidation of meat products, thereby affecting their quality. Natural antimicrobial agents, such as carvacrol, tea polyphenols, catechins, rosemary extract, nisin, and lysozyme, can be used in combination to effectively preserve the safety of meat products throughout their shelf life.

**Table 7:** Effects of combined biotechnologies and non-thermal sterilization technologies on microorganisms.

Physical Technology	Processing Parameter	Biotechnology	Dosages	Target of an Action	Consequences of Action		Medium of Action	Bibliography
Radiographic irradiation	3 kGy	Chitosan solution	1.5%	*-*	The total colony count did not exceed 3.0 log CFU/g in 15 d.	-	Pork	[129]
cold nitrogen plasma (CNP)	(500 W, 120 s)	Lemongrass essential oil	5 mg/mL, 30 min	*Listeria monocytogenes*	*Listeria monocytogenes* population decreased by 2.80 log CFU/g.	Shelf life extended by 4 days.	Pork	[130]
Radio-frequency heating (55 °C)	27.12 MHz, 6 kW	Citric acid and potassium bicarbonate mixture	0.5 per cent citric acid and 1.5 per cent potassium bicarbonate	*Escherichia coli*, *Aerobic bacteria*	*Escherichia coli* and aerobic bacteria plate counts were reduced by more than 4 log CFU/mL.	-	Ground beef (80/20; lean/fat)	[131]
Modified atmosphere packaging	40% CO_2_:60% N_2_	Ag/LDPE	Ag NPs (0.5% or 1%, *w*/*w*)	TVC, *psychrotrophic bacteria*,	The growth rate of psychrotrophic bacteria was slowed down by 16.67 percent.	Shelf life extended to 8 days.	Chicken breast fillets	[132]
Critical carbon dioxide combined with high power ultrasound (SC—CO_2_ + HPU)	25 MPa, 46 °C and 10 min	Saline	0.85%	*Escherichia coli*	Reduced colony count 3.62 ± 0.20 log CFU/g.	Storage 20 days.	Cured ham	[133]
LED	405 nm, 19.2 J/cm^2^	Riboflavin (vitamin B2)	50 μM	*Listeria monocytogenes*	Reduced by 6.2 log CFU/mL.	-	Smoked salmon	[134]
High hydrostatic pressure (HHP)	300 MPa/5 min after tumbling, 600 MPa/3 min for final product	KCl	50 percent partial replacement of NaCl	*-*	-	Shelf life extended by 60 days.	Ready-to-eat (RTE) chicken breast	[135]
Oppressive	600 MPa, 180 s	NaCl	1.5%	*Listeria monocytogenes*	Decrease of 2.49 and 7.29 logarithms.	-	Chicken meat mince	[136]
EAP	2.2 kHz and 8.4 kVpp, 15 min	Clove oil	1.0%	*Escherichia coli O157:H7*	Reduction of more than 7 log CFU/g.	-	Beef jerky	[137]
Air conditioning	MAP, 70% CO_2_, 30% N_2_	Chitosan solution	1 g/100 ml	*Pseudomonas*, lactic acid bacteria, and *Enterobacteriaceae*	*Pseudomonas* spp. control samples were 3.3 log CFU/g lower and *Enterobacteriaceae* populations remained unchanged between days 4 and 14 of storage (~1.0 log cfu/g).	Shelf life extended by 9 days.	Chicken breast	[138]
Modified atmosphere packaging (MAP)	20% CO_2_, 80% N_2_	*Ziziphora clinopodioides essential oil and lysozyme*	ZCEO (0.3%) *lysozyme* (400 μg/g)	*Listeria monocytogenes*	Storage 13 days *Listeria monocytogenes* 2.05 log CFU/g.	-	Balkan-style fresh sausage	[139]
High hydrostatic pressure	400 MPa for 10 min	*entertain LM-2*	2560 AU/g	*L. monocytogenes*, *S. enteritidis*	Prevents the growth of *Streptococcus enterocolitica*, below the detection limit throughout the storage period at 4 °C.	Extending the shelf life to above 90 days.	Ready-to-eat sliced vacuum-packed cooked ham	[140]
Vacuum packaging	-	EO (CA, CI, and TH)	2%	*Pseudomonas aeruginosa*	Reduces microbial populations by up to 4 to 6 log colony forming units (CFU)/g.	-	Chicken work	[141]
Low-voltage electric fields	15 cm, 3000 V, 50 Hz, −1 ± 0.5 °C	Compound preservatives	4% *Lysozyme* + 2% *Nisin* + 0.75% *Eugenol*	*Bacillus subtilis*, *Pseudomonas*	The absorbance values were reduced by 30.16% and 44.58%, respectively.	-	Mytilus edulis	[14]
High-pressure processing	400 MPa, 10 min	Spice extracts	0.05% clove + 0.05% cinnamon extracts	TVC, LAB, *B. thermosphacta*, and *C. perfringens*	TVC was significantly reduced to less than 3 log CFU/g in the 12 day treatment group, and no LAB was detected.	-	Low-salt Sausage	[142]
MAP	80% N_2_, 20% CO_2_	Aronia melanocarpa, Chaenomeles superba, and Cornus mas leaf extracts	5% *v*/*w*	TVC, LAB, and *Enterobacteriaceae*	TVC was significantly reduced to less than 3 log CFU/g in the 12 day treatment group, and no LAB was detected.	-	Pork	[143]
Electron-beam Irradiation	4 kGy	Leek extract on the quality	0.5%, 1.0%	*Escherichia coli*, mold	No mold or *Escherichia coli* were detected.	-	Pork Jerky	[144]

## 5. Conclusions

This paper summarizes the methods of microbial inactivation in meat products using non-thermal physical and biotechnological techniques. Each sterilization technique has its unique advantages and can achieve its intended purpose to some extent; however, it is also constrained by various external factors. Non-thermal physical sterilization techniques have a lesser impact on the organoleptic quality and nutritional content of food and are less likely to cause contamination. However, they often require specialized environments and equipment and are associated with high energy consumption. Moreover, the inactivation of microorganisms may lack stability when environmental conditions fluctuate significantly or when multiple microbial species are present. Bio-sterilization technology is characterized by high safety, the extensive use of biological resources, rich biodiversity, and adaptability; however, it may be influenced by factors such as extraction efficiency, processing costs, and flavor impact.

Optimizing the synergistic combinations of physical inhibition methods and natural inhibitors can broaden the inhibition spectrum and enhance the killing efficacy against different types of microorganisms. This approach can simultaneously reduce the treatment intensity required by a single method and mitigate the potential impact on food quality. Building upon the use of natural bacteriostatic agents, such as plant extracts, in modified atmosphere packaging (MAP), we are delving deeper into the integration of physical bacteriostatic methods with natural bacteriostatic agents. For example, in modified atmosphere packaging, aside from adding plant extracts, microwave treatment can be integrated. Microwaves are capable of rapidly heating the materials. They can not only kill microorganisms to a certain degree but also facilitate the more effective penetration of plant extracts into the food, thereby enhancing the antibacterial effect. The application of optimized combination technologies for foodstuffs with different biological characteristics is studied in depth across various food systems. For foodstuffs rich in oils and fats, research focuses on how to prevent the inactivation of natural bacteriostatic agents in an oily fat environment and the influence of physical bacteriostatic methods on the oxidative stability of oils and fats. Through these studies, precise and personalized combined antibacterial solutions will be provided for the preservation of different foods.

Compared with heat treatment, non-thermal sterilization methods exhibit obvious energy-saving characteristics. For example, ultraviolet sterilization involves using ultraviolet radiation energy to destroy the nucleic acids of microorganisms, achieving the purpose of sterilization. The operation process does not require heating; only a small amount of electricity is consumed to power the ultraviolet lamp, greatly reducing energy consumption. Pulsed electric field sterilization disrupts the cell membranes of microorganisms through the instantaneous application of a high-voltage electric field. Additionally, its energy consumption is substantially lower than that of traditional thermal sterilization methods. By using these non-thermal sterilizing techniques, energy consumption is decreased, environmental pressures associated with energy use are lessened, and sustainable growth is promoted. Natural bacteriostatic agents also possess the property of environmental friendliness. These agents are derived from natural resources such as plants, animals, or microorganisms and are generally biodegradable in the natural environment, causing less environmental pollution. It aligns with contemporary society’s pursuit of a green and environmentally friendly production paradigm and lifestyle. This technology is deserving of further promotion and application, enabling consumers to enjoy high-quality and safe meat products with a sense of reassurance.

With the ongoing development and in-depth study of sterilization technology based on the principle of ‘fence technology’, future research should focus on combining various sterilization methods to improve sterilization efficiency while minimizing flavor and nutritional losses. Additionally, research should aim to develop more efficient and safer microbiological control strategies to manage spoilage and harmful microorganisms during meat processing, thereby enhancing meat quality and ensuring food safety.

## Figures and Tables

**Table 5:** Effects of compound antibacterial agents and compound coating films on microorganisms.

Antibacterial Method	Culture Condition	Target of an Action	Consequences of Action		Medium of Action	Bibliography
The amount of chitosan and tea polyphenols added is 3:1.	4 °C	*Mesophiles*	Growth was reduced by approximately 2.0 log CFU/g.	The expiry date extends the shelf life by 6 days.	Pork	[92]
Novel bioactive sponge mats composed of oxidized bacterial cellulose and chitosan-gum Arabic microcapsules loaded with cinnamon essential oil	4 °C	*S. Staphylococcus aureus*, *Escherichia coli*	Day 10 TVC less than 6 log CFU/g.	Shelf life extended from 4 to 10 days.	Meat	[93]
Highly absorbent antibacterial chitosan-based aerogels	4 °C	*Listeria monocytogenes*, *Staphylococcus aureus*, *E. coli*, and *S. typhimurium*	The diameters of the inhibition zones of *Escherichia coli*, *S. typhimurium Salmonella*, *Listeria monocytogenes*, and *Staphylococcus aureus* increased to 21.65 ± 0.58, 23.35 ± 0.64, 21.86 ± 0.89, and 22.15 ± 0.53 at CuNPs solution (60 μL), respectively, mm.	Shelf life is 14 days.	Pork	[94]
Tannic acid-grafted chitosan coating on the quality (TA-g-CH)	4 °C	*Pseudomonas*	The TVC of pork coated with TA + CH and TA-g-CH at the end of the storage period did not exceed acceptable limits.	Microbiological growth extended the shelf life of pork samples by 6 to 9 days.	Pork	[95]
Effects of chitosan coating with green tea aqueous extract	0 °C	Mesophilic, psychrotrophic	There was a 49.4% and 41.4% reduction in mesophilic and psychrophilic growth, respectively, in the treated group compared to the blank group on day 25 (*p* < 0.05).	-	Pork chops	[96]
Psyllium EmF + 1.0% chitosan	4 °C	*Listeria monocytogenes*	-	Shelf life extended to 15 days	Beef	[97]
Ethylcellulose/gelatin-carboxymethyl chitosan bilayer films doped with Euryale ferox seed shell polyphenol	3~5 °C	*L. monocytogenes*	-	Still has food value after 9 days	Cooked beef and cooked chicken	[98]
Thymol and carvacrol at 0.4 percent and 0.8 percent (*w*/*w*).	4 °C	*Pseudomonas*, *Brochothrix thermosphacta*	*Pseudomonas* reduced to 0.9^−1^ log; *Brochothrix thermosphacta* reduced by 1.1–1.6 logs.	Shelf life extended by 6 days	Corned beef	[99]
0.1% ZEO (Artemisia multiflora essential oil) + 0.2% GSE (grape seed extract)	8 °C	Mesophiles, lactic acid bacteria	Mesophiles and lactic acid bacteria were the most sensitive and membrane-tolerant groups, with reductions of 0.1–1.1 and 0.1–0.7 log cycles, respectively, and a 1.23 log reduction in TVC on day 9 of refrigeration.	-	Instant salami	[100]
0.4% chitosan + 0.02% ε-polylysine + 0.2% ascorbic acid	3 °C	Total viable count (TVC)	After 12 days of storage did not exceed the standard.	Shelf life extended by 6 days	Pork chunks	[101]
Sea buckthorn pomace extract SPF-6–esterified potato starch film	25 °C	*Escherichia coli*, *Listeria monocytogenes*, *Staphylococcus aureus*, *Salmonella*	After 13 months the TVC value reached only 3.67 log CFU/g	-	Beef jerky	[102]
Olive leaf extract (OLE)-gelatin films	23 °C	*L. monocytogenes*	Reduced *L. monocytogenes* growth.	-	smoked salmon	[103]
Chitosan nanoparticles of cinnamon essential oils (CE-NPs) 527 nm	4 °C	*Pseudomonas*	*Pseudomonas*, *Lac-tic Acid Bacteria*, and *Enterobacteriaceae* end-of-treatment colony counts were 5.48, 5.15, and 3.20 log CFU/g.	Shelf life can be extended to 15 days.	Pork	[104]
Sea tangle extract	10 °C	*Enterobacteriaceae*	Eight weeks of storage without exceeding limits.	-	Pork ham	[105]
Lysozyme and Chinese liquor	25 ± 2 °C	*S. aureus*	Total *Staphylococcus aureus* reduced to 2.8 log CFU/g.	-	Dry fermented sausage	[106]
Paeonia japonica (Makino) Miyabe, Takeda, Rhus chinensis Mill, Paeomia suffruticosa, Psidium guajava, Nelumbo nucifera, and Ecklonia cava	21 °C	*E. coli*, *Listeria monocytogenes*, and *Salmonella* spp.	Reduced *E. coli* levels by more than 99.9% after 8 days of storage and slowed the growth of *Listeria monocytogenes* and *Salmonella* spp. by more than 80% after 14 days.	-	Sausages	[107]
Tapioca starch active nanocomposite films and their antimicrobial effectiveness	4 °C	*Listeria monocytogenes*	A reduction of 1 to 2 log CFU/cm^2^ was observed over 10 days	Shelf life is 10 days.	Ready-to-eat chicken meat	[108]
3% chitosan/low-density polyethylene composite film with essential oil of Xiaquan grass	4 °C	*Staphylococcus aureus*, *Bacillus cereus*, *Escherichia coli*, and *Salmonella enteritidis*	-	Shelf life extended to 13 days.	Chicken breast	[109]
Konjac glucan/chitosan antimicrobial composite film with oregano essential oil microcapsules	0–4 °C	*Pseudomonas putida*, *P. fluorescens*, and *Pseudomonas aeruginosa*	10% KGM/CTS inhibition zone size was 10.7 ± 0.58 mm for *Pseudomonas putida*, 9.00 ± 0.00 mm for *P. fluorescens*, and 13.7 ± 0.58 mm for *Pseudomonas aeruginosa*.	The shelf life was extended by 3 days.	Pork	[110]
Nano-encapsulated chitosan film with garlic essential oil	4 °C	*Pseudomonas*	The value is 3.3 colony forming units/g for 50 days.	Storage up to 7 days.	Sausages	[111]
Bacterial cellulose membranes containing streptococcal lactate	4 °C	*Listeria monocytogenes*	The 14 day final count was significantly (*p* < 0.05) lower by approximately 3.4 log CFU/g.	-	Frankfurter sausage	[112]
25 μg/mL Streptococcus lactis and 62.5 μg/mL carvacrol.	4 °C	*Listeria monocytogenes*	Inhibited bacterial growth and increased the doubling time from 15.01 to 23.35 h.	-	Bolognese sausage	[113]
Fabrication of high-stability active nanofibers encapsulated with pomegranate peel extract using chitosan/PEO	4 °C, 25 °C	*Escherichia coli O157:H7*	The number was reduced to 2.96 and 5.80 log CFU/g, respectively.	-	Beef	[114]
A novel fish gelatin film incorporated with protocatechuic acid	4 °C	Total viable counts	Reduced TVC by approx. 0.40–1.68 log_10_ CFU/cm^2^	-	Beef	[115]
Chitosan film containing green tea extracts (CGT-film)	4 °C	aerobic bacteria, yeasts, mold, and lactic acid bacteria	The 12 day total viable count was 5.24 log CFU/g.	-	Pork sausage	[116]
Encapsulation of Phlorotannin in alginate/PEO blended nanofibers mixing ratio 50:50:10 (SA/PEO/Ph)	4 °C, 25 °C	*streptococcus enteritis*	Decreased from 2.92 log CFU/g at 4 °C to 6.27 log CFU/g at 25 °C.	-	Chicken meat	[117]
Chitosan coatings incorporated with free or nano-encapsulated paulownia tomentosa essential oil	4 °C	*Pseudomonas* spp.	*Pseudomonas* spp. counts were maintained below 5 log CFU/g and LAB counts were reduced by 2.61 log CFU/g (*p* < 0.05).	Storage 16 days.	Ready-to-cook pork chops	[118]
Curcumin—cinnamon oil nano emulsion/pectin coating	4 °C	Aerobic bacteria, *Psychrophiles*	The counts of CCNC-coated aerobic bacteria and Psychrophiles were reduced by 97.8% and 99.5% at 12 days, respectively.	Shelf life extended to 12 days.	Chicken fillet	[119]
0.5% Cinnamaldehyde (CV) or muscimol (TM) teriyaki sauce	4 °C	*Escherichia coli*, *Salmonella*, and *Listeria monocytogenes*	The strain was completely inactivated by cold pickling and could not be recovered	-	Corned beef	[120]
Effects of nanoemulsion-based active coatings with a composite mixture of star anise essential oil, polylysine, and nisin	4 °C	*Escherichia coli*	-	Shelf life extended from 8 to 16 days.	Ready-to-eat Yao meat products	[77]
Chitosan-silver nanoparticles	4 °C	*Escherichia coli*, *S. typhimurium*	1000 and 2500 mg/g antimicrobial activity 14.47 and 9.00 × 10^4^ log CFU/g.	-	Minced meat	[121]
Nanoemulsion with star anise essential oil, polylysine, and nisin	4 °C	*E. coli*	*E. coli* growth was reduced by approximately 1 log CFU/g.	Shelf life extended from 8 to 16 days	Ready-to-eat Yao meat	[77]

**Table 6:** Effects of combined physical techniques on microorganisms.

Physical Technology	Technical Parameters	Culture Condition	Target of an Action	Consequences of Action		Medium of Action	Bibliography
LEEB irradiation with superchilled	0.2 MeV;8 kGy	−1.0 ± 0.5 °C	*Weissella*, *Carnobacterium*, and *Lactobacillus*	On the 10, 20, and 30 day of storage *Weissella*, *Carnobacterium*, and *Lactobacillus* abundance decreased by 9.29%, 29.98%, and 14.02%, respectively.	Shelf-life of pork to (at least) 30 days	Pork	[122]
Ozone treatment and vacuum packaging	2 mg/L, 5, 10 mg/L	4 ± 1 °C	Lactic acid bacteria	Lactic acid bacteria reached 2 log CFU/g on days 14–16 for the 2 mg/L treated samples and did not reach 7 log CFU/g within 16 days of the 5 or 10 mg/L ozone treatments.	The shelf life of chicken thigh samples treated with 2 mg/L, 5, or 10 mg/L ozone was extended by 4, 6, and 6 days, respectively (*p* < 0.001).	Drumstick	[123]
Electron beam irradiation and air conditioning	40% CO_2_/60% N_2_, 4 kGy irradiation	4 °C	-	-	Shelf life extended by 28 days.	Duck in sauce	[124]
High Pressure Vacuum	600 MPa, 3 min	4 °C	TAC	The TAC of the treated product remained below 0.5 log CFU/g for a 30 day storage period.	-	Frankfurters	[125]
Pulsed intense light with UV irradiation	Cured meat thickness 3 mm, 10 cm from the pulsed light source, 11 cm from the UV light source, and irradiated for 5 min.	10 °C	-	The total colony count decreased from 1.5 × 10^7^ log CFU/g to 5.6 × 10^4^ log CFU/g.	-	Preserved meat	[126]

## Data Availability

No new data were created or analyzed in this study. Data sharing is not applicable to this article.
